# Extracellular Vesicles as Mediators of Environmental and Metabolic Stress Coping Mechanisms During Mammalian Follicular Development

**DOI:** 10.3389/fvets.2020.602043

**Published:** 2020-11-19

**Authors:** Samuel Gebremedhn, Asghar Ali, Ahmed Gad, Radek Prochazka, Dawit Tesfaye

**Affiliations:** ^1^Animal Reproduction and Biotechnology Laboratory, Department of Biomedical Sciences, Colorado State University, Fort Collins, CO, United States; ^2^Department of Animal, Rangeland and Wildlife Sciences, Mekelle University, Mekelle, Ethiopia; ^3^Institute of Animal Physiology and Genetics of the Czech Academy of Sciences, Liběchov, Czechia; ^4^Department of Animal Production, Faculty of Agriculture, Cairo University, Giza, Egypt

**Keywords:** extracellular vesicles, environmental stress, follicular development, metabolic stress, intrafollicular cells

## Abstract

Extracellular vesicles are evolutionarily conserved nano-sized phospholipid membraned structures and released from virtually all types of cells into the extracellular space. Their ability to carry various molecular cargos (mRNA, miRNA, proteins, and lipids) from one cell to the other to exert functional impact on the target cells enables them to play a significant role in cell to cell communication during follicular development. As the molecular signals carried by extracellular vesicles reflect the physiological status of the cells of origin, they are expected to mediate any effect of environmental or metabolic stress on the follicualr cells and the growing oocyte. Recent studies have evidenced that reproductive cells exposed to various environmental stressors (heat and oxidative stress) released extracellular vesicles enriched with mRNA and miRNA associated with stress response mechanisms. Moreover, the metabolic status of post-calving cows could be well-reflected in the follicular extracellular vesicle's miRNA profile, which signified the potential role of extracellular cellular vesicle molecular signals in mediating the effect of metabolic stress on follicular and oocyte development. In the present review, the potential role of extracellular vesicles in mediating the effect of environmental and metabolic stress in various reproductive cells and oocytes are thoroughly discussed Moreover, considering the importance of extracellular vesicles in shuttling protective or rescuing molecular signals during stress, their potential usage as means of targeted delivery of molecules to mitigate the effect of stress on oocytes are addressed as the focus of future research.

## Introduction

Throughout their lifespan, including embryonic development, growth, maturity, and aging, animals are exposed to various environmental (heat, cold, oxidative, chemicals, and others) and endogenous stressors. Recurrent environmental and metabolic stress pose significant risk and disruption of the reproductive physiology. Over the past five decades, the intensive selection practices of dairy cows for higher milk yield have resulted in tremendous success in increasing the net milk yield. However, the increase in milk production has resulted in a concomitant reduction in fertility traits (1), which increases the likelihood of culling cows with lower fertility, causing a reduction in the total amount of milk produced and farm profitability (2). This is attributed to the negative correlation of production traits and vulnerability to environmental and metabolic stress. Environmental and metabolic stress factors induce changes in the steroidogenic capacity of follicles, mainly reduce estradiol production in the dominant follicles, which impairs ovulation associated processes (3, 4). Oocytes obtained from follicles exposed to both environmental and metabolic stressors have lower developmental competence (5), accompanied by reduced ability to fertilize and develop to the blastocyst stage and establish a pregnancy (6). Exposure to both environmental and metabolic stress impair the communication between the oocyte and the surrounding follicular fluid. The bi-directional communication between follicular cells and the oocyte is mainly carried out either directly through gap junction or secretion of paracrine and autocrine molecules (7). The recent advancements in the discovery and characterization of extracellular vesicles (EVs) released from various cell types have provided an additional layer into the existing and well-known mechanism of cell-to-cell communication (8). It has been reported that EVs are important in shuttling bioactive molecules (mRNAs, miRNAs, and proteins), which could reflect the physiological status of the originating cells (9, 10). The bioactive molecular content of EVs released from cells subjected to a variety of environmental and metabolic stressors are distinct and divergent compared to the EVs obtained from the unstressed cells counterparts (11). For instance, cells subjected to heat stress released heat shock proteins via EVs (12), and upon uptake by recipient cells, it resulted in the modulation of the immunological responses of the recipient cells (13). Similarly, supplementation of cells with EVs obtained from cells challenged with oxidative stress transported protective messages to the recipient cells upon subsequent exposure to oxidative stress (14). Recently, we have reported the rescuing effect of EVs from H_2_O_2_ treated bovine granulosa cells by modulating the NRF2 signaling pathways (15). Experimental models involving the application of heat, oxidative, and metabolic stress factors on cultured granulosa could provide insights into the possible association of intrafollicular communications with the oocyte. Identifying the conserved and stressor-specific bioactive molecules shuttled via EVs would be beneficial in addressing the specific stress-associated decline in oocyte maturation. The present review highlights the role of EVs in modulating metabolic and environmental stresses. The importance of EVs in intrafollicular communication and ovarian physiology, the rescuing and protective impact of EVs against subsequent stressors are thoroughly discussed.

## Intrafollicular Communication Between the Follicular Cells and the Oocyte

During the course of folliculogenesis, continuous bi-directional communication between the oocyte and its encircling cumulus cells, granulosa cells, and theca cells, to exchange an oocyte and somatic cell factors, is indispensable for the ovulation of a developmentally competent oocyte, that can undergo through fertilization and the processes of embryogenesis (16). The gap junction is the main mechanism of the cross-talk between the oocyte and the follicular cells, which involves the formation of the protein family connexins known for their variability in the permeability, which in turn aids in the transportation of molecules of lower molecular weight lower than 1 KDa in size, which are ions, metabolites, and amino acids between the oocyte and the follicular somatic cells (17). The follicular microenvironment has two physical barriers that separate the oocyte from the follicular cells, the follicular fluid-filled antrum and the zona pellucida (ZP). Nevertheless, the constant flow of information between the oocyte and the follicular cells is maintained through the transzonal projection (TZP), irrespective of the physical barriers (18). The cumulus cells, which are located adjacent to the oocyte are the primary origin of the TZP. In addition to the cumulus cells, which are proximate to the oocyte, cumulus cells positioned at distant layers from the oocyte also are reported to stretch a few TZPs (19). The number of TZP in the growing oocyte is significantly higher, which signifies a growing quest for somatic cells factors for its maturation and the oocyte factors for the specialization of the surrounding somatic cells (20), and signifies the stimulatory effect of the oocyte on the surrounding somatic cells to generate more TZP. The study further showed that the oocyte-secreted GDF9 stimulates the surrounding cells to generate more TZP through the SMAD signaling pathway. In a separate study, oocyte from mouse model deficient in GDF9 showed to have a malformation of TZP (21). It has been evidenced that, at the tip of the TZP a split is formed and EVs have been detected at the tip of the splits, which signifies the importance of TZP in shuttling EVs between the somatic cells and the oocyte (22).

The formation of an antrum cavity filled with a serum-like fluid exudate called the follicular fluid is the defining phenomenon of the later stages of mammalian follicular development (23). The follicular fluid, which is a mixture of proteins, lipids, and nucleotide and ions, secreted from the surrounding somatic cells and the oocyte, and blood plasma, which cross the follicular barrier through the capillaries of the theca cells, serves as an enclosed microenvironment for the maturing oocyte (24). The follicular fluid is a source of gonadotropins, growth factors, enzymes, and proteins and the biochemical composition of the follicular fluid could also be a reflector of the pathophysiological conditions of the corresponding follicle and predictors of the oocyte quality (25). The follicular fluid in the antrum segregates the oocyte from the mural granulosa cells and theca cells, which creates a conducive microenvironment for normal development of the oocyte, and further facilitate the exchange of cellular messages between them (24). The physical separation between the oocyte and the follicular cells leads to the formation of two distinct subpopulations of granulosa cells. The outer layers of granulosa cells will be separated from the granulosa cells layers adjacent and surrounding the oocyte, which becomes cumulus cells. Nevertheless, the communication between the oocyte and follicular cells is still maintained mainly through paracrine signaling. Here, each subpopulation of granulosa cells is being exposed to the FSH and oocyte secreting factors, at differential gradients (26). For instance, the interaction of the oocyte secreted factors like the GDF9 and BMP15 with the FSH and growth factors like the IGF1 is crucial in promoting the proliferation and differentiation of the granulosa cells and the cumulus cells, respectively (27).

## Extracellular Vesicles as Means of Intrafollicular Communications

Extracellular vesicles (EVs) are evolutionarily conserved nano-sized, phospholipid bilayer membraned structures of varying sizes released from virtually all types of cells through the exocytosis process into the extracellular space (8). EVs are reported to be present throughout an organism's biological fluids providing pleiotropic functions (28). Due to their heterogeneity in the size, shape, membrane protein, and the originating cells (29), EVs can broadly be categorized as microvesicles, exosomes, and apoptotic bodies. Microvesicles are generated by the outward budding of the plasma membranes with irregular shapes and a size range of 100–1,000 nm. Exosomes are the smallest fraction of EVs, with a diameter ranging from 30 to150 nm and formed from the intraluminal vesicles (ILV), with the multivesicular bodies (MVB) and released to the extracellular space upon fusion with the plasma membrane (30). The largest fractions of EVs are the apoptotic bodies, with a diameter range of 1 to 5 μm, which arise from the highly regulated cellular disintegration during apoptosis (31). EVs are also differentiated based on the molecular bioactive molecular content they encapsulated. Exosomes and MVs encapsulate an array of cytoplasmic contents including RNAs, proteins, and lipids (32), while apoptotic bodies are equipped with nuclear components and cellular organelles (33). The presence of specific membrane-associated proteins including the tetraspanins, the CD63, CD81, and CD9, and other proteins including ALIX and TSG101 is the hallmark of EVs (34). Studies have suggested that the encapsulated bioactive molecules within EVs can exert phenotypic changes and modulate the expression of genes in the secreting and nearly and distantly located recipient cells (35). Cells actively select the amount of EVs and the type of bioactive molecules to shuttle depending on the cellular physiological and environmental conditions, like stress and diseases (36). Upon reaching the target cells, EVs can interact with their target cells either through EVs membrane protein interaction and the cellular receptors or through the dissolution of the EVs protein by the enormous protease activity in the extracellular space making the EVs release their content and act on the receptors of the target cells. Besides, the membrane of the EVs could be fused with the membrane of the recipient cells to release the content of the EVs and incorporate it into the cellular content (37).

EVs are abundantly present in virtually all reproductive biological fluids including follicular fluid (38), oviductal fluid (39, 40), uterine fluid (41), amniotic fluid (42), and spent cell culture media (15) and embryos culture media (43). EVs carry molecular conservatories including mRNA, miRNAs, and proteins, which indicate the physiological status of the originating cells (9, 10). EVs are reported to be present in the follicular fluid of bovine (38, 44, 45), equine (46), human (47) and are essential in the transport of RNAs, miRNAs, and protein to recipient cells during follicular development. In bovine, the uptake of follicular EVs is reported to be associated with the alteration transcript abundance in the recipient oocytes (48), and granulosa cells (38), and enhanced the cumulus expansion during *in vitro* oocyte maturation (44), the rate of development of *in vitro* fertilized oocytes to the blastocyst stage (49) and proliferation of granulosa cells (50). It is not only the amount of EVs in the biological fluid that varies depending on the physiological status of the reproductive organ, but also the molecular cargo carried by these EVs. For instance, a small growing follicle not only contains a higher concentration of EVs in the follicular fluid, but also a large set of EV-coupled miRNAs, which evidenced the molecular dynamics during the processes of oocyte growth. Intriguingly, EVs isolated from follicular fluid of small follicles have better potential to support oocyte maturation cumulus expansion with inter-species wide conserved manner (44).

One of the key factors that contribute to the expansion of the cumulus cells by promoting the response to FSH is an oocyte secreted factor named the cumulus expansion enabling factor (CEEF) (51). The CEEF has been speculated to be a combination of oocyte secreted and several other proteins, including the oocyte-secreted GDF9 (52) and the TGFBB1 (53), which have been studied due to their positive impact on cumulus expansion. In support of this notion, denaturation of oocyte maturation conditioned media at higher temperature or treatment with proteinase K completely inhibited the impact of CEEF in inducing the cumulus expansion (54). This signifies the fact that oocyte secreted proteins can be released as components of the CEEF into the extracellular space. The experiment further examined the size of the protein in the conditioned maturation media by using a 100-kDa filter membrane and it was shown that the flow-through component of the conditioned media did not enhance the expansion of the cumulus cells. This implies that the CEEF could be a protein molecule with higher molecular weight (>100 kDa) (54). However, in another study, it was shown that the 25 kDa TGBB1 protein partially plays the role of CEEF (55), signifying the fact that the CEEF is not a single protein, rather a complex of protein molecules. In addition, the presence and activity of CEEF in follicular fluid was investigated by supplementing follicular fluid obtained from small and large follicles during *in vitro* oocyte maturation (54). In that study, it was shown that the cumulus cells expanded extensively in the presence of follicular fluid from the smaller follicles, contrary to the cumulus cells cultured with follicular fluid obtained from larger follicles. Similarly, maturation media conditioned with mural granulosa cells of smaller follicles resulted in elevated CEEF activity and the expansion of the cumulus cells as opposed to the granulosa cells from the larger follicles. This can be attributed to the higher number of FSH receptors in small follicle granulosa cells as compared to the large ones.

To date, the role of EVs in shuttling the CEEF between the oocyte and the surrounding somatic cells is not reported. However, reports on the differential effect of EVs supplementation from the small and large follicles on cumulus expansion could indicate the potential role of EVs in shuttling the CEEFs between the ovarian somatic cells and gamete and vise versa (44). Nevertheless, further study is needed to profile the proteome content of the EVs derived from the follicular fluid at different stages of the follicular development, which could lead to the identification of either known or novel CEEFs circulating in the follicular environment.

Due to its enclosed microenvironment, the follicle is convenient to study the EV-signaling mechanism, as the source of the EVs and recipient cells can easily be pinpointed. Another advantage of the follicular microenvironment is the remarkable stability of the RNA molecules in the follicular fluid and other reproductive biological fluid, irrespective of the higher nuclease activity, which could be attributed to the encapsulation in EVs (9). This makes the EVs encapsulated RNAs promising molecular tools in the search for the diagnostic markers of various reproductive pathophysiological conditions. For instance, analysis of the EV-mediated miRNAs derived from human follicular fluid and the corresponding serum samples revealed specific miRNAs enriched in the EVs of the follicular fluid with an important role in ovarian functions (47). The study showed the miRNAs specifically enriched in the EVs of follicular fluid including miR-99a, miR-100, miR-132, and miR-218 are involved in the maturation process of the follicles, while the miR-132, miR-212, and miR-214 negatively regulate genes known to encode inhibitors of follicular maturation (47). Supplementation of oocytes with EVs from follicular fluid has been shown to alter the transcript abundance of the recipient oocyte and played enhancing the competence role of the oocyte to reach the blastocyst stage (49). In an attempt to determine the impact of follicular fluid EVs on the physiology and morphology of the cumulus-oocyte complexes (COCs) and the associated changes in the gene expression, follicular fluid EVs derived from small and large bovine antral follicles were supplemented to COCs (44). The study further showed that the EVs from the follicular fluid of smaller follicles harbor more bioactive molecules and pose a tremendous positive impact on the expansion of the COCs and enrichment of the COCs expansion marker genes, Ptgs2, Ptx3, and Tnfaip6 in both bovine and mouse. Interestingly, the study verified that supplementation of bovine follicular fluid EVs into mouse COCs induced the expansion of cumulus cells (44). This signifies the fact that the evolutionary conservation of the EVs functions and opens a room for inter-species alternatives for the remedies in reproductive pathophysiological conditions. EVs of the follicular fluid carry miRNAs reported to be involved in key pathways related to oocyte maturation, such as the WNT, TGFß, MAPK, and ErbB signaling pathways (46, 47). It is also worth noting the developmental competence of bovine oocytes is closely associated with the EV-coupled miRNA profile of the corresponding follicular fluid. (38). The study further revealed the higher number of miRNAs being released via EVs from follicular fluid, which surrounds the immature oocytes compared to the EVs derived from follicular fluid encompassing mature oocyte. This is in agreement with the previous finding, which highlighted that the smaller follicles release more EVs into the follicular fluid compared to the larger antral follicles (56). The study further affirms that the miRNAs content of EVs from the follicular fluid is reported to show variation according to the stage of development. For instance, the expression of miR-204, miR-92b, miR-328a-3p, miR-424e-3p, and miR-450a is reported to progressively increase, while the expression of miR-19a-3p and miR-335 showed a progressive decline in response to the increase in the size and growth of follicles. Follicles of variable size have been reported to have distinct sets of miRNAs (56), which could have a differential impact on the proliferation of granulosa cells (50). Similarly, in mare, the follicular fluid EVs obtained from the preovulatory and the mid-estrous stage follicles are enriched with miR-372, miR-27b, and miR-382 and these miRNAs have an inhibitory role on genes like the Inhibitor of DNA Binding/Differentiation 2 (ID2), which are down-regulated in the granulosa cells of mare preovulatory dominant follicles (57). A summary of the EV-mediated transfer of bioactive molecules in reproductive biofluids is indicated in Table 1.

**Table 1:** Summary of EV-mediated release of bioactive molecules and the associated biological functions.

**Reproductive biofluid/media**	**Bioactive molecules released in EVs**	**EV-associated biological functions**	**Species**	**References**
Follicular fluid	miRNAs^1, 2, 4, 5, 6, 13^, transcripts associated with cumulus expansion^3^, C-type natriuretic peptide (CNP) and natriuretic peptide receptors subtype 2 (NPR2)^7^, transcriptome^8, 9, 10^, proteome^11^	Oocyte developmental competence^1^, Post-partum negative energy balance^2^, cumulus expansion^3^, follicular growth and maturation^4, 5, 6^, oocyte meiosis progression^7^, Alteration of bovine oviductal epithelial cells transcriptome^8^, Association the transcriptome of EVs with the neighboring mural granulosa cells^9^, enhancement of meiotic resumption^10^, Mediate heat-stress associated gene expression in oocyte^11^, Improvement of oocyte competence and survival under heat shock^12^, predictors of IVF outcomes and pregnancy success^13^	Bovine^1, 2, 3, 5, 7, 8, 10, 12^, Equine^4^, Human^6, 13^, Porcine^9^, Feline^10^	^1^(38), ^2^(45), ^3^(44), ^4^(46), ^5^(56), ^6^(47), ^7^(58), ^8^(59), ^9^(60), ^10^(61), ^11^(48), ^12^(62), ^13^(63)
Oviductal fluid	Proteins^1, 2, 3, 4^, miRNAs^2, 4^	Embryo-oviduct cross-talk^1^, gamete-oviduct interations^2^, Change in the Phospholipid Composition of *in vitro* developed Bovine Embryos^3^, Hormonal impact of the estrous cycle on EVs secretion^4^, regulation of sperm motility and survival^5^, regulation of polyspermy during porcine *in vitro* fertilisation^6^, improvement of birth rates after embryo transfer^7^	Bovine^1, 2, 3^,Porcine^4, 5, 6^Mouse^7^	^1^(39), ^2^(64), ^3^(65), ^4^(66), ^5^(67), ^6^(68), ^7^(69)
Granulosa cells spent culture media	miRNAs^1^, heat-stress associated transcripts (HSP70, HSP90, GRP78, and GRP94)^1^ and oxidative Stress-associated transcripts (NRF2, PRDX1, CAT, and TXN1)^2^	The protective impact of EV-associated miRNAs against heat stress^1^, rescuing role of EVs against oxidative stress^2^	Bovine^1, 2^	^1^(70), ^2^(15)

*Superscript numbers correspond to the references listed in the last column of the table*.

## Impact of Environmental and Metabolic Stress on Follicular Development and the Involvement of EVs in Mediating the Stress Coping Mechanisms

Among the determinant factors that contribute to the decline of female fertility is exposure to both environmental and metabolic stresses. Among the environmental stressors, oxidative (15) and heat stress (71) is reported to impair follicular development and function of the follicular cells. Moreover, metabolic stressors, which enhance the accumulation of non-esterified fatty acids in the circulation are also reported to impair the steroidogenic function of the follicular cells (72).

### Oxidative Stress

Among the stress-inducing factors that arise from environmental and physiological insults is oxidative stress, which is a phenomenon, where the amount of cellular ROS production overwhelms the intrinsic production of scavenging antioxidants (73, 74). The ROS are the most abundant forms of intermediates generated during the oxygen consumption process (75) and are involved in oxidative stress (76). Even though a moderate level of ROS is beneficial for normal progression of cellular functions (77), excessive production can surpass the natural antioxidant system and this creates an unfitting environment for female reproduction (78). During ovarian follicular development, there is a complex relationship between the production of ROS and the counterbalancing antioxidants in a stage-dependent manner (79). For instance, during the preovulatory stage, the post-LH inflammatory precursors generate excessive ROS, which is important to induce ovulation (80). Among the free radicals that lead to the excessive accumulation of intracellular ROS and subsequent cellular damage, the H_2_O_2_ has a longer cellular half-life and can enter into the nucleus (81). Thus, it is a widely used free radical for the induction of oxidative stress under *in vitro* experiments in various cell types. Reports showed that H_2_O_2_ induces granulosa cell apoptosis by regulating the ROS-JNK-p53 pathway (82). Bovine granulosa cells treated with H_2_O_2_ accumulated a significantly higher amount of ROS accompanied by reduced activity of mitochondria and elevated expression of the stress-related transcription factor NRF2 and its downstream antioxidant transcripts (*CAT, PRDX1*, and *TXN1*) (15). One of the factors responsible for the impaired cellular functions of *in vitro* developed oocyte is the generation of oxidative stress from the suboptimal maturation condition, which could contribute to the lower success of *in vitro* embryo production (83). The extent that oocyte is affected by oxidative stress during *in vitro* maturation process depends on the intrinsic antioxidant properties and how fast this is replenished during the maturation process (84). Interestingly, oocytes with intact cumulus cell mass are endowed with higher antioxidant activity compared to denuded ones (84).

The involvement of EV-mediated molecule transfer in response to oxidative stress has been previously reported. The EVs released from mouse mast cells transport protective RNA messages to the recipient cells upon coincubation against subsequent exposure to oxidative stress (14). In our previous experiment, we showed that EVs released from granulosa cells treated with H_2_O_2_ are enriched with NRF2 and antioxidant enzymes (CAT and TXN1), signifying the fact that EVs could partly reflect the cellular stress conditions considering the presence of stress-associated transcripts both in the cells and the released EVs (15). In the same study, the co-incubation of EVs obtained from H_2_O_2_ challenged granulosa cells rescued the recipient granulosa cells by reducing the accumulation of intracellular ROS. This signifies the fact that stress associated EVs contain molecular signals, which could play a protective or rescuing role against subsequent stress. Nevertheless, the abundance of the oxidative stress-associated transcripts like the NRF2 and its downstream antioxidants in the recipient cells was elevated and subsequently, reduce intracellular ROS accumulation (15). This could be because of the fact that pretreatment of cells with H_2_O_2_ before the supplementation of EVs has already activated the transcription of the NRF2 signaling pathway. In a separate study, EVs obtained from cells exposed to H_2_O_2_ were supplemented to recipient cells before subsequent H_2_O_2_ challenge showed to have a protective impact in the recipient cells against subsequent oxidative stress (14). This demonstrates the fact that the timing of stress primed EVs supplementation in relation to the timing of the subsequent H_2_O_2_ exposure determines the transcriptional activation pattern in the recipient cells.

### Heat Stress

Environmental heat stress during summer seasons is a dominant stressor in the dairy and beef industry that leads to impairment in various reproductive processes including oocyte maturation, embryo development, gonadotropin secretion, ovarian follicular growth, steroidogenesis, development of corpus luteum, and uterine endometrial responses (4, 85). Summer heat damage has a long-lasting effect on dairy cows and a period of 2–3 estrous cycles are required to fully recover from heat stress-induced damages (4). Exposure of oocytes to high temperature during the maturation period resulted in impairment in the rearrangement of their microtubules and microfilaments, damaged spindle apparatus, an increased proportion of oocytes arrested at metaphase, the nuclear maturation (5) and resulted in a lower rate of fertilization (6). Similarly, oocytes collected during the summertime are reported to have lower mitochondrial distribution compared to oocytes collected during wintertime, which was accompanied by the induction of oxidative stress and apoptosis (86). Heat stress also impairs the function of granulosa cells, which in turn compromises ovarian function and the developmental competence of the accompanied oocytes (87). We demonstrated the deleterious effect of heat stress on granulosa cells using an *in vitro* cell culture model, in which exposure of cells to elevated temperature resulted in reduced cellular proliferation, increased apoptosis, increased ROS accumulation, and reduced mitochondrial activity (88). Granulosa cells respond to heat exposure by activating the heat shock proteins (HSP) family and the unfolded protein response (UPR) (89, 90). Among the heat shock protein family, the HSP70 is reported to be significantly elevated in granulosa cells exposed to heat stress (71).

The cellular reserve of the HSPs is also reported to be present in the extracellular space. For instance, cells subjected to heat stress are reported to release HSPs into the extracellular space via EVs (12, 91, 92) and are involved in modulating the immunological responses of the recipient cells (13). Similarly, cells exposed to heat stress are reported to release the HSPs into the extracellular space via EVs, which could be a reflector of the cellular heat stress conditions (93). Cells exposed to heat stress are reported to release EVs that could pose a bystander effect on recipient neighboring cells. The bystander effect can be explained by the reduced rate of apoptosis and DNA damage in the untreated recipient cells (94). Interestingly, the study showed that the cells supplemented with EVs from heat-stressed cells became more resistant to subsequent heat stress compared to the untreated counterparts. We recently experimented on the EVs-mediated transfer of protective signals against heat stress in bovine granulosa cells (70). Data showed that granulosa cells subjected to heat stress (42°C) released a significantly higher number of EVs to the culture media compared to the cells kept under normal temperature (37°C). Moreover, the EVs of the heat-stressed granulosa cells were enriched with HSP70, HSP90, and SOD1 and tend to have more GRP78 and GRP94 compared to the EVs of untreated granulosa cells. Interestingly, the miRNA profile of the EVs showed that the heat-stressed EVs are enriched with miR-1246, which is also previously reported to be abundantly expressed in the circulation of heat-stressed Holstein Frisian cows (95). A recent study in bovine has evidenced that supplementation of both the whole follicular fluid and isolated EVs from follicular fluid reversed the damage of the heat stress on the oocyte and improved the cleavage and blastocyst rates (62). Interestingly, supplementation oocytes with only follicular EVs have a better impact than the whole follicular fluid in terms of improving cleavage and blastocyst rate. This signifies that the EVs play a major role in reversing the damage incurred by heat stress on the oocytes.

### Metabolic Stress

The intensive selection for high milk yield in the past decades has resulted in a significant increment in the amount of milk per lactation per cow. However, a concomitant reduction in the fertility traits of high yielding dairy cows was also observed (1). The decline in fertility traits is associated with early lactation post-partum negative energy balance (NEB) (96). Follicles obtained from post-partum cows under NEB undergo several unfavorable metabolic changes that affect the developmental competence of oocytes (97, 98). Similarly, granulosa cells of cows under NEB exhibited lower expression of genes linked to Vitamin A and D metabolism, suggesting the detrimental effect of metabolic stress on bovine follicular development (99). Among the major metabolic changes in high-producing dairy cows is the elevation of free fatty acids (FFA) in the circulation, which are released from adipose tissues during the early post-partum period (100). Besides the changes in the composition of post-partum follicular fluid, cows under NEB are characterized by the elevated concentration of the non-esterified fatty acid (NEFA) and b-hydroxybutyrate concentrations (BHB) during the first 2–4 weeks post-partum and start to decline from the 6th week post-partum both in the follicular fluid and serum (101). The concentration of palmitic (C16:0), stearic acid (C18:0), and oleic acid (C18:1) are reported to be elevated in the serum of cows under NEB (102). Under *in vitro* culture conditions, supplementation of individual and pooled NEFA components (C16:0 and C18:0) to bovine granulosa cells at a dose as low as 150 mM induced apoptosis and reduced proliferation of cells, which signifies the toxic effects of NEFA accumulation during the post-partum period on the follicular cells in bovine (72).

We recently examined the miRNAs content of EVs derived from follicular fluid of cows under divergent metabolic status post-calving and results showed a massive down-regulation of miRNAs in follicular fluid EVs of cows under NEB (45). Based on the miRNA fingerprints carried by follicular EVs, cows under positive energy balance post-partum closely resembled heifers than cows under NEB conditions. Moreover, the oocytes derived from these cows during early post-partum showed altered epigenetic profiles compared to those derived during the late post-partum period (103). Interestingly, the study identified around 33,000 differentially methylated regions (DMRs) were found to be specific to early-post-partum and these were located within genes involved in metabolic, carbon metabolism, and fatty acid metabolisms. However, the association of EV-coupled molecular signatures with an altered epigenome profile of oocytes needs further investigation.

## Dynamics of EV-mediated miRNAs in Follicular Cells in Response to Environmental Stress

**Figure 1:**
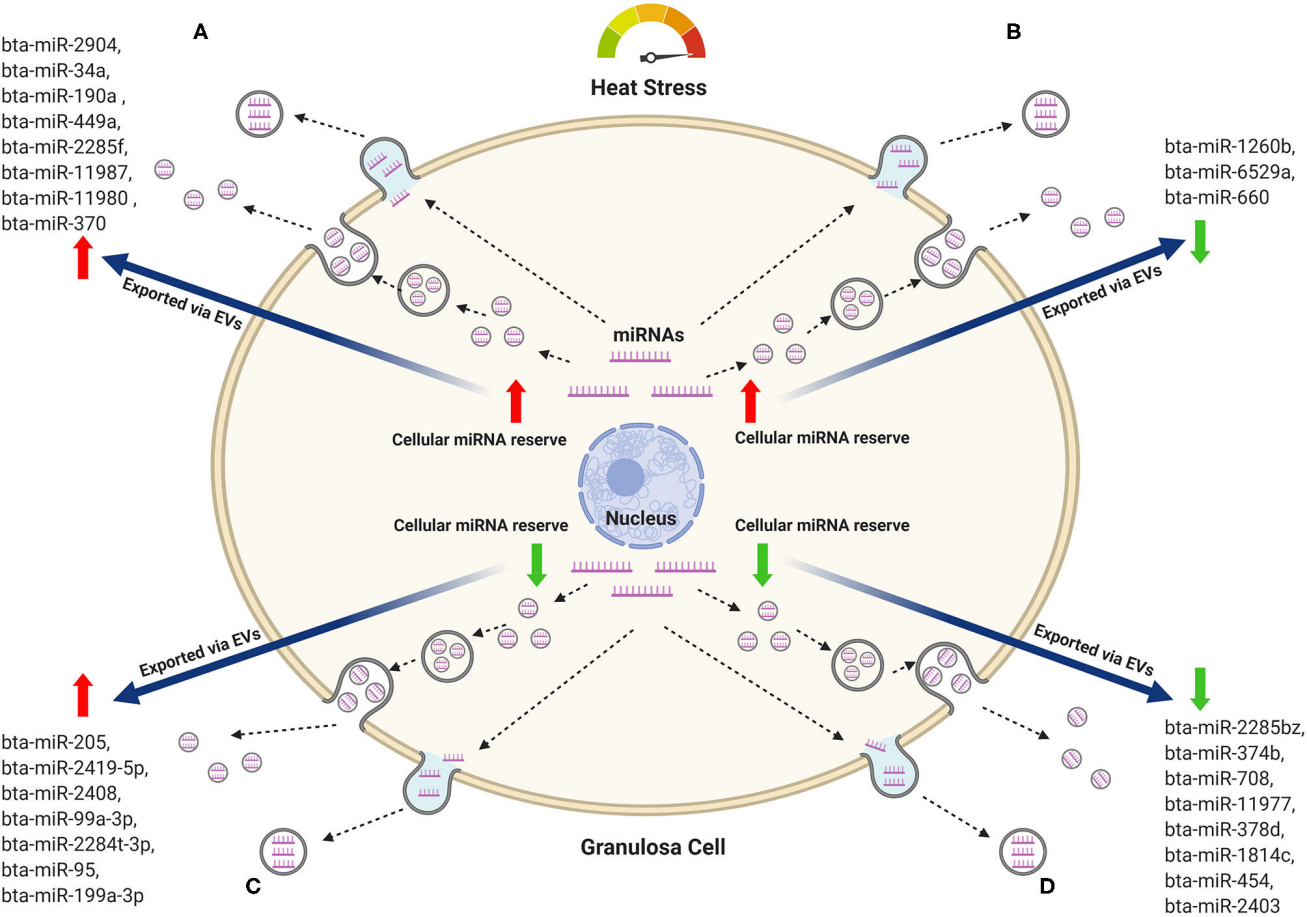
Hypothetical model of the miRNAs dynamics in bovine granulosa cells and the corresponding EVs in response to heat stress. Representative miRNAs that belong to each pattern are listed. **(A)** MiRNAs with increasing trend both in the cellular reserve and in the released EVs. **(B)** MiRNAs increased in the cellular reserve and declined in the released EVs. **(C)** MiRNAs depleted in the cellular reserve but increased in the released EVs. **(D)** MiRNAs depleted in both the cellular reserve and the released EVs. Red arrows represent an increasing trend, while green arrows represent a declining trend.

One of the fundamental aspects of EVs-mediated transfer of bioactive molecules is the balance between the molecules remained in the cells and those released into the extracellular space. EVs released into the body fluid could indicate the level of intracellular hemostasis (104). Similarly, cells could also release EVs as a mechanism to remove toxicants from cells (105). For instance, EV-mediated release of b-catenin through EVs led to the reduced intracellular reserve of the ß-catenin pool, which in turn downregulate the intracellular WNT signaling pathway (106). Nevertheless, it is arguable whether the intracellular reserve of certain bioactive molecules will be reduced irrespective of its enrichment in the corresponding EVs. In addition, the exposure of cells to either environmental or metabolic stress could potentially have a unique pattern of release of a selected bioactive molecule. To address this, we performed a comparison of the miRNAs profile in granulosa cells and the corresponding EVs in relation to exposure to heat stress (70). MiRNAs were sorted according to their expression pattern in the heat-stressed group, where miRNAs with positive or negative fold changes are considered as up or down-regulated miRNAs, respectively. Accordingly, four different scenarios of miRNAs expression in granulosa cells and EV-mediated release were observed namely, (A) miRNAs upregulated in both the cells and the corresponding EVs, (B) miRNAs upregulated in the granulosa cells, but downregulated in the corresponding EVs, (C) miRNAs downregulated in the granulosa cells, but upregulated in the corresponding EVs, and (D) miRNAs downregulated in both the granulosa cells and the corresponding EVs. The mechanisms of these patterns and the selective release or maintaining of these miRNAs could either be a reflector of the cellular homeostasis or as mechanisms to reduce the cellular reserve of selected miRNA, which are detrimental to the survival of cells during the exposure to environmental or metabolic stressors. Contrary to this, the miRNAs, which are preferentially enriched in the cells as opposed to the corresponding EVs or vice-versa could be beneficial to the cells to cope with the exogenous stress and contribute to the enhanced development of stress resistance. A hypothetical model that describes the dynamics of miRNA abundance in the cellular reserve and released EVs as a response to heat stress is indicated in Figure 1.

## The Future Implication of EVs in Mammalian Female Fertility During Environmental and Metabolic Stress

The importance of EVs as molecular cargo in mediating mammalian follicular development has been characterized in several reproductive biofluids. Nevertheless, the functional role of these EVs and their cargo molecules are not fully understood. Considering the important roles of EVs in shuttling the protective and rescuing signals in follicular cells, it would highlight the potential usage of EVs as a means of molecule delivery, which could be utilized for future applications to mitigate the effect of stress on oocytes and embryo development. The molecular characterization of the cargo of EVs with a potential impact on oocyte and embryo development could lead to the discovery of molecular markers for the development of stress-associated infertility treatment strategies. Therefore, characterizing the content of EVs released from granulosa cells and oviductal epithelial cells after exposure to environmental and metabolic stress could provide useful insight about the survival mechanisms of reproductive cells and possible usage of these EVs as supplementation into the oocyte maturation and embryo culture medium to enhance stress-coping mechanism during oocytes maturation and embryos development. This will be relevant to address the issue of the qualitative and quantitative decline in the outcome of the *in vitro* production of embryos, which arises from the various stress-inducing factors under *in vitro* environment including the oxygen tension and culture media constituents compared to their *in vivo* counterparts.

## Author Contributions

SG and DT: literature searches. SG: writing. AA: illustration. DT, AA, AG, and RP: editing. DT, AG, and RP: approving and submission. All authors contributed to the article and approved the submitted version.

## Conflict of Interest

The authors declare that the research was conducted in the absence of any commercial or financial relationships that could be construed as a potential conflict of interest.
